# Japanese students’ perception of their learning from an interprofessional education program: a qualitative study

**DOI:** 10.5116/ijme.50e5.e29a

**Published:** 2013-01-17

**Authors:** Takami Maeno, Ayumi Takayashiki, Tokie Anme, Eriko Tohno, Tetsuhiro Maeno, Akira Hara

**Affiliations:** 1 School of Medicine, School of Medicine and Medical Sciences, University of Tsukuba, Ibaraki, Japan; 2 School of Nursing, School of Medicine and Medical Sciences, University of Tsukuba, Ibaraki, Japan; 3 Total Health Evaluation Center Tsukuba, Ibaraki, Japan

**Keywords:** Qualitative research, interprofessional education, empowerment, interprofessional problem-based learning, Japan

## Abstract

**Objectives:**

The aim of this study was to explore students’ perception of their learning from the interprofessional program implemented in Japan where the implementation and evaluation of interprofessional education is behind that of western countries.

**Methods:**

We conducted a qualitative research of opinions of students who participated in the interprofessional program implemented in the University of Tsukuba. The participants were 105 medical, 65 nursing, and 35 medical science students. At the completion of the program, we asked that the participants write their opinion on what they gained by participating in the program. From their responses, significant descriptions were extracted, coded by content, and then grouped into subcategories. These subcategories were then separated into main categories based on their emergent themes.

**Results:**

The main categories identified were such as “understanding of medical professionals,” “interprofessional work,” “holistic care,” “communication,” “sharing,” and “empowerment.”

**Conclusions:**

The categories extracted in our study, for the most part, matched previous studies, suggesting that the program helped students enhance their understanding of interprofessional work. Although the Japanese health care system and medical education system are different from those of western countries, this suggests that the benefits of interprofessional education in Japan will be similar to those of western countries.

## Introduction

With the aging of the population, an increase in chronic disorders, and other social changes, health professionals are increasingly required to provide continuous care for patients who face complex problems. In the fields of health, medicine, and welfare, a variety of care services are provided by these specialists. It is almost impossible for anyone health professional to address the complex problems of patients and implement appropriate, continuous care alone. In order to improve the quality of patient care, collaboration between health and social care professionals has become vital.^1^^,^^2^Interprofessional education (IPE) is an important element in the adoption of the basic skills required for interprofessional care.^3^ IPE has been promoted around the world as an integral part of undergraduate education.^4^ In fact, the 2009 revised edition of “Tomorrow’s Doctors,” published by the General Medical Council,^5^ which provides the outcomes of and sets standards for undergraduate medical education in the UK, sets the goal for medical professionals: “Learn and work effectively within a multi-professional team.”As for Japan, although the team approach to health care has also been included in the model core curriculum in undergraduate medical education, until a decade ago, no university or college in Japan had incorporated a large-scale IPE program into their educational curriculum in order to facilitate the collaborative learning process of multi-disciplinary students. Since the year 2000, an increasing number of colleges have introduced interprofessional education into their curriculum.^6^In respect of the evidence regarding the effectiveness of IPE, a Cochrane review of IPE published in 2008^7^ whose objective was to assess the effectiveness of IPE interventions, showed that IPE in the clinical workplace produced positive outcomes in a number of areas. These areas were patient satisfaction and safety in the emergency department,^8^^,^^9^ management of care delivered to domestic violence victims,^10^ and better delivery of care by mental health professionals.^11^The Best Evidence Medical Education (BEME) review published in 2007^12^ provided evidence from 21 papers. Two-thirds of the studies were on pre-qualification education. Outcomes were classified into six categories: level 1: reaction, level 2a: modification of perception and attitudes, level 2b: acquisition of knowledge and skills, level 3: behavioural change, level 4a: change in organization practice, and level 4b: benefit to patients/clients. The review showed positive results with leaners mainly in levels 1, 2a, and 2b.Although these studies reported some positive outcomes, because of the heterogeneity of IPE initiatives and methodological limitations, generalization is difficult about the effectiveness of IPE. Continued evaluations of IPE are warranted.In particular, because IPE has only recently been introduced in Japan, little is known about its effectiveness there. Japan’s cultural background, health care system, and medical education system are different from those of western countries. What type of program is most effective and what points should be stressed when developing an IPE program in Japan have yet to be adequately clarified. It is important to evaluate the program in order to develop a successful program suited for Japan.In 2006, The School of Medicine and Medical Sciences, University of Tsukuba, introduced an IPE program. The University of Tsukuba, established in 1973, is located in Ibaraki Prefecture in Japan, approximately one hour from Tokyo via train. The School of Medicine and Medical Sciences consists of three schools for health professionals: the School of Medicine, the School of Nursing, and the School of Medical Science. The School of Medicine trains physicians; the School of Nursing, nurses; and the School of Medical Science, medical technicians and medical scientists.We conducted a qualitative study to explore the students’ perception of their learning from an IPE program. We used a qualitative approach for this study as we would like to begin examining what is the nature of the students’ perception of their learning. This is due to the fact that there is currently little understanding of the effects of Japanese IPE programs. Qualitative approaches are best used for discovering the answers to ‘why’, ‘how’ or ‘what is the nature of …’ type questions whereas quantitative research focuses on answering the question ‘how much’.^13^^,^^14^ To this end, we conducted a qualitative study to explore the students’ perception of their learning from an IPE program.

## Methods

### Outline of the Care Colloquium (teamwork training course)

In 2006, the School of Medicine and Medical Sciences, University of Tsukuba, Ibaraki, Japan, introduced the Care Colloquium (teamwork training course), an IPE program, designed to encourage medical, nursing, and medical science students to learn the importance of interprofessional work. The program is a one-week long compulsory course. Each year, about 100 third-year medical students, 80 fourth-year nursing students, and 40 fourth-year medical science students participate in the program. Medical students participate in IPE prior to their clinical training, whereas nursing and medical science students take part after completing their clinical training.The program uses problem-based learning (PBL) to teach the importance of interprofessional work and collaboration. Students from the three schools are placed in small groups of seven to eight. Each group contains at least one person from each profession: 3-4 medical students, 2-3 nursing students, and 1-2 medical science students. We use case scenarios with 3-4 groups using the same scenario. The contents of all scenarios require something of each profession in the care of the patients and their families. These scenarios include themes such as home care for a patient with amyotrophic lateral sclerosis, a patient with diabetes, a patient with breast cancer, and home care for a post-stroke patient. Using the viewpoints of each health care professional, they discuss how they should best cooperate with each other to resolve the problems of the patients and their families.The program is a one-week long program. The schedule consists of an orientation, an icebreaker, two core times, two question times, group discussion, and a general presentation meeting. At the orientation, the coordinators explain the goal of the program to students. We show three terms as key words: care, empowerment, and interprofessional work. Core time is given for group discussion with a tutor from one of the three schools. Group work is dedicated to group discussion without a tutor. Question time is the venue for students to ask scenario writers case-related questions. At the end of the week, in the general presentation meeting, each group gives a presentation of what they had discussed regarding resolving the problems of patients and their families.

### Participants

The participants were the students of the three schools who were enrolled in the Care Colloquium in 2007: 105 medical students (mean age 21.2±0.9, female 35.2%), 65 nursing students (mean age 23.1±3.4, female 100.0%), and 35 medical science students (mean age 22.3±0.8, female 73.5%)- a total of 205 students. As the Care Colloquium is part of the required curriculum of the three schools, all students in the requisite year of study in their school participated.

### Data collection

On the final day of the 2007 program, we conducted a self-administered, open-ended questionnaire. We asked that the participants write a concise response to the question “what did you gain by participating in the Care Colloquium?”

### Data analysis

The analysis of the written responses was performed by qualitative analysis to generate emergent themes. Researchers from different scientific fields (two medical, one nursing, and one medical science program coordinator) conducted the analysis. We used Steps Coding and Theorization (SCAT) for analysis.^15^^,^^16^ SCAT was developed as an easily accessible qualitative data analysis method. The background of methods is from the grounded theory approach. The analysis method consists of generative coding and theorization. This method is applicable for analyses of open-ended questionnaire responses. The written responses were first reviewed by the first and second authors, and significant sections were extracted. These were then coded by content, keeping the original opinion intact. Next, these were reviewed and divided into subcategories based on this coding, and each given a title. These subcategories were then consolidated into main categories based on their emergent themes, and given a title. The first and second authors worked together to analyze and title these themes and categories. The third author and the fourth author read the data separately and checked the quality of the themes identified by the first and second authors. The final step of SCAT is developing a story-line and offering theories that weave together the themes and constructs. A diagram was then created by the four authors to indicate the relationships within the main categories.

### Ethical considerations

When the questionnaire was given to the participants, they were all informed that no personally identifiable information would be used in the results, that there was no penalty for non-participation, and that the questionnaire had no bearing on their grade.Because the results would be utilized to improve the program, formal research ethics approval was not obtained. However, due to the findings and potential benefit to other training establishments, the decision was made to share the results via publication.

## Results

A total of 96 medical, 64 nursing, and 35 medical science students responded to the survey. The response rate was 95.1%. From their open-ended responses, 356 descriptions were extracted and coded by content, and grouped into 48 subcategories based on this coding. These were then grouped into 14 main categories based on their emergent themes. The main categories based on their emergent themes identified were understanding of health professionals, multiple viewpoints and perspectives, review of one's own ideas, awareness of one's own specialty, interprofessional work, holistic care, knowledge of social resources, communication, group discussion, sharing, empowerment, personality, learning using specific scenarios, and impressions.In the paragraphs that follow, square brackets ([…]) denote main categories, angle brackets (<…>) denote subcategories, and double quotation marks (“…”) denote the representative descriptions. Common themes described by a large number of students of the three specialties were [interprofessional work], [understanding of health professionals], and [multiple viewpoints and perspectives].

Table 1 shows one part of their representative descriptions. Their descriptions on [interprofessional work] included the following:

**Table 1:** Participant perceptions by category

Categories /subcategories	Representative descriptions	Number of Total	Descriptions medicine	Nursing	Medical science
Understanding of health professionals					
Variety of professionals	I learned that a wide variety of professionals are involved in health care (medicine).	8	7	1	
Understanding of other health professionals	I now understand the positions and roles of other health professionals (medical science).	48	24	21	3
Importance of understanding other health professionals	I am required to understand the roles of professionals of different specialties so that I will be able to provide patients with advice (medicine).	10	5	4	1
Common basic principles	I realized that health professionals from different fields share a common awareness: “for patients”, “for the families of patients”, and “to help patients and their families live a normal daily life” (nursing).	3	1	2	
Multiple viewpoints and perspectives					
Variety of viewpoints	Although we used the same scenario as an information source, we had completely different viewpoints depending on our own health profession (nursing).	27	12	14	1
Different approaches	I had a valuable experience communicating with nursing and medical science students. I learned from them a variety of patient approaches (medicine).	4	3		1
Different ideas	It was a good opportunity for me to listen to other health professionals and learn many different ideas (medical science).	12	2	6	4
Broad perspectives	It was like I had discovered a new world (medical science).	12	1	7	4
Necessity of having multiple viewpoints	I realized that an interprofessional team is important for providing patient care that is based on more than one perspective (medicine).	1	1		
Review of one’s own ideas					
Review of one’s own ideas	I only have focused on lesions when I saw a patient (medicine).	5	4		1
Awareness of one’s own specialty					
Awareness of one’s own specialty	As a health professional, I realized that I am required to obtain more knowledge and expertise in my specialty to cooperate with specialists from other health care fields (medicine).	24	11	11	2
Interprofessional work					
Experience and impressions of interprofessional work	I was able to experience care services provided as interprofessional work (nursing).	2	1	1	
What acquired from implementing interprofessional work	It was interesting to design therapeutic plans as a care team (medicine).	4	2	2	
Understanding of inter- professional work	An experience of implementing patient-centered care as a team helped me achieve a better understanding of interprofessional care (nursing).	15	10	4	1
Effects of interprofessional work	Collaboration within an interprofessional team allows them to address more difficult challenges (nursing).	20	7	9	4
Importance of interprofessional work	It is difficult for a single health care specialist to provide care in the best interests of a patient. I realized the importance of treating patients as a team while collaborating with a variety of health professionals (medicine).	35	18	12	5
Difficulties of interprofessional work	It was not easy for care specialists from different fields to work together to treat one patient (nursing).	4	1	3	
Cooperation	It is necessary for a variety of health professionals to cooperate with an increased awareness as a medical specialist (medical science).	1			1
Assignment of roles	I learned the approaches required to empower patients by making the best of different health professionals (medicine).	2	1	1	
Equality in relationships	I used to have a biased view that the leader of an interprofessional team should be a physician. However, I learned by participating in this course that all health care specialists should establish relationships on an equal footing with each other (nursing).	2		2	
Holistic care					
Importance of care	I have come to pay more attention to follow-up care in addition to disease treatment.	11	8		3
Holistic care	I recognized the necessity of providing a patient with holistic care, including his/her relationship with the family, and its difficulty (medicine).	3	2		1
Customized care for individual patients	The provision of care services customized to each patient is important (medicine).	2	1		1
A trusting relationship with patients	We should consider a child patient and his/her parents as “team members" (medicine).	5	5		
Necessity of taking into consideration the background of a patient	We should provide care while communicating with patients and taking into consideration their familial and other backgrounds (medicine).	3	2		1
Problems other than diseases	Disease is not the only problem that a patient has (medicine).	1	1		
Care from the standpoint of a patient	It is difficult but important to express opinions and ideas from the standpoint of a patient (medical science).	8	4	1	3
Patient-centered care	I was able to think about what is necessary for patient-centered care (nursing).	8		6	2
Care goals	I recognized the importance of seeking the most appropriate care for a patient (nursing).	3	2		1
Knowledge of social resources					
Knowledge of social resources	I realized that there were a variety of health care professions, facilities, and services (medicine).	2	1	1	
Communication					
Experience and impressions of communication	I was able to communicate with students of other specialties very well (medicine).	3	1	1	1
Attitudes in communication	It is important to accept the ideas of others while respecting their opinions (nursing).	2	1	1	
Methods for communication	I learned to develop a good relationship within a team through communication and improve discussions (nursing).	1		1	
Importance of communication	I realized that communication with other professionals will be important when I work as a professional in the future (medical science).	5	2	2	1
Difficulty in communication	I found it difficult to convey my thoughts to those who have studied in fields different from mine (nursing).	1		1	
Group discussions					
Experience of group discussions	I expressed my opinions as a member of the team in discussions (nursing).	2		1	1
Attitudes in group discussions	I developed the ability to discuss while respecting other health professionals (nursing).	2		2	
Methods for group discussions	I learned to attentively listen to others and express my opinions with appropriate timing (medical science).	4		2	2
Effects of group discussions	I had not had much experience with participating in group work before. Through exchanging opinions with other health professionals, I learned to focus on different aspects of things (medical science).	4		2	2
Difficulty in holding a discussion	It was not easy for care providers of many different specialties to discuss the same subject (medicine).	1	1		
Sharing					
Sharing of ideas	Sharing ideas with each other helped me promote a better understanding (nursing).	7	1	6	
Sharing of knowledge	Sharing ideas and knowledge with each other is important (medicine).	2	1	1	
Sharing of goals	I learned the importance of health care specialists from different fields sharing their policies and goals (nursing).	7	2	5	
Sharing of problems	I was able to address the problems of patients in collaboration with other health care professionals (medicine).	1	1		
Empowerment					
Empowerment	Care does not merely mean helping patients with disabilities. Of utmost importance is enabling them to make the most of their remaining abilities, i.e., empowerment is more important (medicine).	7	3	1	3
Personality					
Personality	I realized that sociality as a physician and as a person is very important (medicine).	4	3		1

Note: Responses to the questionnaire were collected from 96 medical, 64 nursing, and 35 medical science students. Then, 356 descriptions were extracted, coded by content, and grouped into 48 subcategories. These were grouped into 14 main categories based on their themes.

* “I realized the importance of treating patients as part of a team while collaborating with a variety of health professionals,”* (Y3 medical student, male)

and

*“Collaboration within an interprofessional team allows us to address more difficult challenges.”* (Y4 nursing student, female)

Some students experienced difficulty in providing interprofessional care:

*“I learned about the difficulties care specialists from different fields face when working together to treat a patient.”* (Y4 nursing student, female)

Regarding [understanding of health professionals], some students commented as follows:

*“I learned that a wide variety of professionals are involved in health care,”* (Y3 medical student, female)

and

“*I now understand the positions and roles of other health professionals.”* (Y4 medical science student, female)

Regarding [multiple viewpoints and perspectives], their descriptions included statements such as,

*“Although we used the same scenario as an information source, we had completely different viewpoints depending on our own health profession,”* (Y4 nursing student, female)

and expressed surprise, for example,

*“It was like I had discovered a new world.”* (Y4 medical science student, female)

Students also described the importance of multiple perspectives:

*“I realized that an interprofessional team is important for providing patient care that is based on more than one perspective”* (Y3 medical student, male).

[Communication], [sharing], and [empowerment] were also common themes extracted from student descriptions of the three specialties. Descriptions regarding [communication] included,

*“I realized that communication with other professionals will be important when I work as a professional in the future,”* (Y4 medical science student, female)

and

*“I found it difficult to convey my thoughts to those who have studied in fields different from mine”* (Y4 nursing student, female).

There were also descriptions on the importance of [sharing]:

*“Sharing ideas and knowledge with each other is important,”* (Y3 medical student, male)

and

*“I learned the importance of health care specialists from different fields sharing their policies and goals.”* (Y4 nursing student, female)

Descriptions regarding [empowerment] included,

*“Care does not merely mean helping patients with disabilities. Of utmost importance is enabling them to make the most of their remaining abilities, i.e., empowerment is more important.”* (Y3 medical student, female)

[Awareness of one’s own specialty] was another common descriptive category for the students of the three specialties. Descriptions included,

*“As a health professional, I realized that I am required to obtain more knowledge and expertise in my specialty to cooperate with specialists from other health care fields.*” (Y3 medical student, female)

Regarding differences between specialists, there was no description by nursing students in the category of [review of one’s own ideas], whereas medical and medical science students commented,

*“I only have focused on lesions when I saw a patient,”* (Y3 medical student, male)

and

“It is more important to pay attention to a patient rather than a disease.” (Y3 medical student, female)

Related to the category of [holistic care], a large number of medical and medical science students expressed their opinions, but nursing students offered fewer descriptions. A medical science student wrote,

*“During my four years of learning in the field of laboratory tests, I only focused on the causes of diseases, laboratory data, and treatment, placing little emphasis on the background of a patient as well as mental and other support.”* (Y4 medical science student, male)

Others said,

*“Now, I also place importance on post-treatment care.”* (Y3 medical student, male)

and

*“I recognized the necessity of providing a patient with holistic care, including his/her relationship with the family, and its difficulty.”* (Y3 medical student, female)

As for [personality], one medical student stated,

*“I realized that sociality as a physician and as a person is very important.”* (Y3 medical student, male)

**Figure 1:**
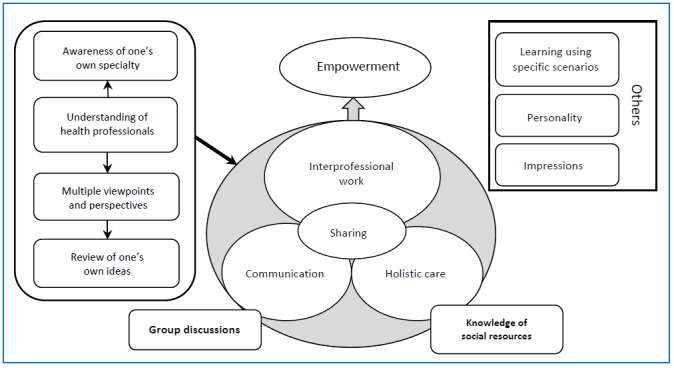
Relationships among main categories

Descriptions by nursing students included,

“I used to have a biased view that the leader of an interprofessional team should be a physician. However, I learned by participating in this course that all health care specialists should establish relationships on an equal footing with each other.” (Y4 nursing student, female)

which shows their recognition of the importance of <equality in relationships>. Figure 1 shows the relationships between the main categories. A better [understanding of health professionals] helped students increase an [awareness of their specialty], recognize the necessity of having [multiple viewpoints and perspectives], and encouraged the [review of one’s own ideas]. Through these experiences, the students learned the importance of [interprofessional work]. They also learned the importance of [holistic care] for patients living in the community. To provide [holistic care], collaboration among different care professionals, [interprofessional work], is essential. They also realized the importance of [communication] and [sharing] in collaboration, and recognized the need for [empowerment]. [Interprofessional work] promotes the empowerment of not only patients and their families but also of the students themselves.

## Discussion

The students’ perception on their learning from the IPE program included interprofessional work, communication, understanding of health professionals, holistic care, sharing, empowerment, and more. The results suggested that the IPE program had helped Japanese students enhance their understanding of interprofessional work. The results of Thistlethwaite and Moran’s review to answer the question “What are the learning outcomes for health professionals that may only be achieved completely through IPE?” states that the six broad themes of outcomes are: teamwork, roles and responsibilities, communication, learning and reflection, the patient/client, and ethics and attitudes.^17^ The categories extracted in our study, for the most part, matched these themes.A large number of students commented on the importance of interprofessional work and communication regardless of their future specialty, which indicates their recognition of its significance as well as of the opportunities to learn and practice it. Previous studies evaluated undergraduate IPE programs with qualitative methods^18^^,^^19^ showed that students felt IPE had the potential for improving communication and enhancing cooperation. A similar observation was found in the Japanese program.A large number of students also commented on the understanding of health professionals. Kilminster et al. ^18^ evaluated the effect of the learning from pre-registration interprofessional workshop with qualitative methods and showed that the participants reported learning on the development of greater awareness of the roles of both other professions and their own. Barker and Oandasan^20^ suggested that knowledge of professional roles is an essential component in IPE initiatives and suggested that understanding professional roles is essential in order to establish collaboration. It might be valuable for the students to understand the roles of health professionals for achieving collaborative practice. The program also might be effective as an educational approach towards promoting professionalism, in that working with other students from different fields encouraged them to review their own professional roles, and to increase the awareness of their own specialty.The students recognized that it is also important for health professionals not simply to provide care services to patients and their families but also to empower them. The belief that empowering of patients is a vital part of patient care,^21^ the students are presented with “empowerment” as one of the keywords during orientation. Recognition of the importance of empowerment is one of the unique characteristics of this program. One improvement that could be made in future programs would be the creation of scenarios and the facilitation of discussions to help students recognize how crucial such empowerment really is.There were some differences in student learning, depending on the specialty. Medical and medical science students learned to think from a nursing perspective, to review a disease-oriented approach, and to recognize the importance of holistic care for patients and their families. Leaning about the ‘care viewpoint’ that is the de facto approach for nurses instead of the ‘cure viewpoint’ that is central to the studies of doctors and medical techniciansprovided them with a fresh perspective. The medical and medical science students viewed it as a valuable experience. The students from each school differed in age, gender, and experience of clinical training. These differences may have some influence on their perception of their learning from IPE.On the other hand, there were few descriptions of differences of opinion or interprofessional conflict. Since health care professionals often experience these problems in real clinical settings, students must also learn to address interprofessional issues. This points to one of the program’s limitations, as its discussion sections are based only on scenarios. Previous studies exploring the effectiveness of IPE programs showed that in a community-based IPE program in Japan, students learned about interprofessional conflict in a clinical setting.^22^ To address these problems, it is important to implement such programs in real clinical settings in addition to learning from a virtual scenario.Ponzer et al. ^23^ showed that the quality of supervision was the most important factor to students’ satisfaction with IPE. The BEME review concluded that staff development is a key influence on the effectiveness of IPE for learners who all have unique values about themselves and others.^12^ Thus, it is necessary to implement staff development for faculty to provide effective facilitation and further the development of effective scenarios.One of the limitations of this study was that as written responses were used, we could not investigate deeply. Further studies should be able to more thoroughly investigate via interviews the questions of “how” students felt towards their IPE experience and “why”.There is only limited information about the longer-term impact of IPE. Hylin et al.^24^ showed that according to a follow-up survey, former healthcare students who underwent undergraduate training on interprofessional work had lasting and positive impressions of it; the results suggest the continuous effects of IPE training. However, there is a lack of long term evaluation of IPE. Evaluation of the long-term effects of pre-registration IPE on the delivery of services and care for patients is still needed.As only a small number of colleges have implemented an IPE program so far, only a few studies of its effectiveness have been made in Japan.^25^ It is essential to evaluate for developing IPE programs in undergraduate education to train health professionals to be able to collaborate and cooperate with a variety of other health specialists on a continual basis.

## Conclusion

In this study, students showed an enhanced understanding of health professionals, interprofessional work, communication, holistic care, sharing, and empowerment. This study covered students’ perception of their learning as a qualitative study. Further studies are required to provide quantitative data on the change in perceptions, attitudes, knowledge, skills and behavior via pre-post studies and the implementation of follow-up investigations.

### Conflict of Interest

The authors declare that they have no conflict of interest.
